# Association Between Diagnostic Delays and Spinal Involvement in Human Brucellosis: A Retrospective Case-Control Study

**DOI:** 10.1093/ofid/ofae357

**Published:** 2024-06-28

**Authors:** Zhongshu Pu, Yiwen Liu, Manling Bai, Tong Ling, Jing Pan, Dengrong Xu, Peijun Dai, Yongping Yan

**Affiliations:** Department of Infectious Diseases, 940th Hospital of Joint Logistics Support Force of the Chinese People's Liberation Army, Lanzhou, China; Department of Epidemiology, Ministry of Education Key Lab of Hazard Assessment and Control in Special Operational Environment, School of Public Health, Air Force Medical University, Xi’an, China; Department of Immunization Program, Wuwei Municipal Center for Disease Control and Prevention, Wuwei, China; Department of Infectious Diseases, Wuwei People's Hospital, Wuwei, China; Department of Hygienic Logistics, 940th Hospital of Joint Logistic Support Force of the Chinese People's Liberation Army, Lanzhou, China; Department of Hygienic Logistics, 940th Hospital of Joint Logistic Support Force of the Chinese People's Liberation Army, Lanzhou, China; Department of Infectious Diseases, 940th Hospital of Joint Logistics Support Force of the Chinese People's Liberation Army, Lanzhou, China; Department of Infectious Diseases, 940th Hospital of Joint Logistics Support Force of the Chinese People's Liberation Army, Lanzhou, China; Department of Epidemiology, Ministry of Education Key Lab of Hazard Assessment and Control in Special Operational Environment, School of Public Health, Air Force Medical University, Xi’an, China

**Keywords:** diagnostic delay, human brucellosis, old age, risk factors, spinal

## Abstract

**Background:**

Spinal involvement is a common but serious complication of human brucellosis. However, information on the risk factors associated with spinal involvement in individuals with brucellosis is limited.

**Methods:**

This retrospective case-control study aimed to determine the potential risk factors associated with spinal complications in inpatients with brucellosis.

**Results:**

During the study period, brucellosis was diagnosed in 377 patients, of whom 108 (28.64%) showed spinal involvement. Those with spinal involvement were significantly older than patients in the control group (mean age [standard deviation], 53.25 [10.48] vs 43.12 [13.84] years, respectively; *P* < .001). The diagnostic delays were significantly longer in patients with spinal involvement than in the control group (mean delay [standard deviation], 11.17 [13.55] vs 6.03 [8.02] weeks; *P* = .001). Age >40 years (odds ratio, 5.42 [95% confidence interval, 2.65–11.05]; *P* < .001) and diagnostic delay >4 weeks (2.94 [1.62–5.35]; *P* < .001) were independently associated with spinal involvement in brucellosis. The lumbar spine at the L3–5 level was the most affected (152 of 249 [61.04%]). Back pain (92 of 108 in case patients vs 21 of 108 in controls; P < .001) and splenomegaly (23 vs 42 of 108, respectively; *P* = .005) differed significantly between the 2 groups.

**Conclusions:**

Age >40 years and diagnostic delay >4 weeks increased the risk of spinal involvement in brucellosis. Therefore, the time from symptom onset to diagnosis should be shortened, using effective measures to reduce spinal involvement risk.

## Teaser

Individuals with spinal involvement were significantly older and had a longer mean diagnostic delay than those in the control group. The risk of spinal involvement in brucellosis increased in patients aged >40 years and having a diagnostic delay >4 weeks.

Brucellosis is one of the most common zoonotic diseases caused by *Brucella* species [1]. Human brucellosis is a major public health concern in several regions, mainly the Mediterranean region, the Middle East, and parts of Central and South America [2]. Brucellosis affects organs with abundant mononuclear phagocytes, such as the liver, spleen, lymph nodes, and bone marrow [3]. Given its unique pathogenic characteristics, it frequently causes various complications. One serious complication is spinal involvement; brucellosis frequently requires spinal surgery and results in functional sequelae [4].

Approximately 2%–53% of patients with brucellosis demonstrate spinal involvement [5]. A retrospective study in Turkey reported that 39% of patients with brucellosis presented with spondylodiscitis [6]. In Southern Tunisia, 22% of patients had spinal brucellosis diagnosed [7]. Furthermore, 8%–20% of patients with brucellosis presented with spinal involvement in high-risk regions in China [8–10]. Brucellar spondylitis typically occurs in men aged >40 years. On average, patients with spinal involvement are significantly older than those without spinal involvement [5, 9, 10].

Spondylitis mostly affects the lumbar spine, followed by thoracic and cervical regions [11]. The most common complaints of spondylitis include fever, malaise, sweating, back pain, and anorexia. Epidural, paravertebral, prevertebral, and psoas abscesses or radiculitis can occur in brucellar spondylitis. Therefore, correct and early diagnosis and immediate treatment are necessary [12]. Radiological imaging is crucial in evaluating the disease process. Although radiography and computed tomography (CT) may provide some information, magnetic resonance (MR) imaging is highly sensitive in detecting early-stage disease and enlargement in epidural and paravertebral areas.

The clinical and radiological findings of spine-involved brucellosis are mostly atypical and difficult to diagnose because of its nonspecific and variable clinical imaging. Brucellosis is diagnosed late in most patients with vertebral osteomyelitis, with a mean diagnostic delay of 12.7 weeks [13]. At present, the association between diagnostic delay and brucellosis spine involvement remains unreported, and the epidemiological features and risk factors for spinal involvement in patients with brucellosis are largely unknown. Hence, this retrospective case-control study aimed to determine the epidemiological features of and risk factors associated with spinal complications in inpatients with a diagnosis of brucellosis.

## METHODS

### Study Design

This retrospective case-control study was conducted with a 1:1 case-control ratio at 2 hospitals in Gansu Province, the most brucellosis-endemic area in China. Patients with brucellosis diagnosed at the 940th Hospital of the Joint Logistic Support Force of the Chinese People’s Liberation Army (PLA) and Wuwei People's Hospital between January 2018 and July 2023 were enrolled; in particular, those in whom radiological or MR imaging showed spinal involvement were included for the case group. For the control group, patients without spinal involvement were randomly selected from the enrolled inpatients during the same period.

### Inclusion and Exclusion Criteria and Definitions

*Brucella* infections were diagnosed based on the positive results of the Rose Bengal plate agglutination test and standard agglutination test results ≥1/100. Isolation of *Brucella* species from blood samples also led to the diagnosis of *Brucella* infections. In this study, clinical, radiological, or MR imaging evidence of inflammation in ≥1 vertebra or discitis was defined as spinal brucellosis, whereas any evidence of inflammation in any joint was defined as osteoarticular involvement. Scrotal or epididymal pain with positive ultrasonographic findings indicated the presence of epididymo-orchitis.

The study excluded patients with lumbar disk herniation of undetermined cause and tuberculous vertebra, those without any symptoms diagnosed via screening, and those with insufficient information. Diagnostic delay was defined as the interval between the onset of brucellosis symptoms and the confirmed diagnosis at the healthcare institution. Moreover, patients with a systemic disease duration of <12 months before hospitalization were classified as having acute-subacute brucellosis, and those with a systemic disease duration of >12 months were classified as having chronic brucellosis.

### Data Collection

Patients’ demographic characteristics (sex, age, ethnicity, and occupation), contact history (exposure to goats or cattle and a family history of brucellosis), clinical manifestations (clinical signs and symptoms), laboratory parameters, and radiological data were obtained from the hospital information system and clinical examinations. For the laboratory analyses, the following parameters were included: complete blood cell count, urinalysis, blood biochemical examination, erythrocyte sedimentation rate, and C-reactive protein level. As for the radiological data, CT or MR imaging of suspicious areas, echocardiography, scrotal Doppler ultrasonography, and other imaging examinations were conducted according to the patient's symptoms. Two experienced clinicians collected all of these data.

### Statistical Analysis

Categorical variables are presented as numbers and percentages, and continuous variables as means with standard deviations. Independent samples *t* test was used for comparing the means of continuous variables from data with normal distribution, whereas the Mann-Whitney *U* test was used for comparing continuous variables from data without normal distributions. For the categorical variables, the proportions were compared using χ^2^ tests. The contingency tables were analyzed using Fisher exact tests.

Stepwise logistic regression analysis was also conducted. Associations with spinal involvement in brucellosis were examined using bivariate analyses. In the bivariate analysis, odds ratios (ORs), 95% confidence intervals (CIs), and *P* values were calculated using χ^2^ or Fisher exact tests. All variables with a *P* value ≤.10 in the bivariate analysis were included in multivariate logistic regression analysis. Forward elimination was conducted using the likelihood ratio test. Logistic regression analysis results are reported as adjusted ORs with 95% CIs. Differences were considered statistically significant at *P* < .05 (2 sided). All statistical data were analyzed using SPSS 22.0 software (SPSS).

### Patient Consent Statement

All included patients provided written informed consent. The study design was approved by the local ethics committee (Ethics Committee of the 940th Hospital of the Joint Logistic Support Force of the PLA; protocol no. 2022KYLL056).

## RESULTS

**Figure 1.**
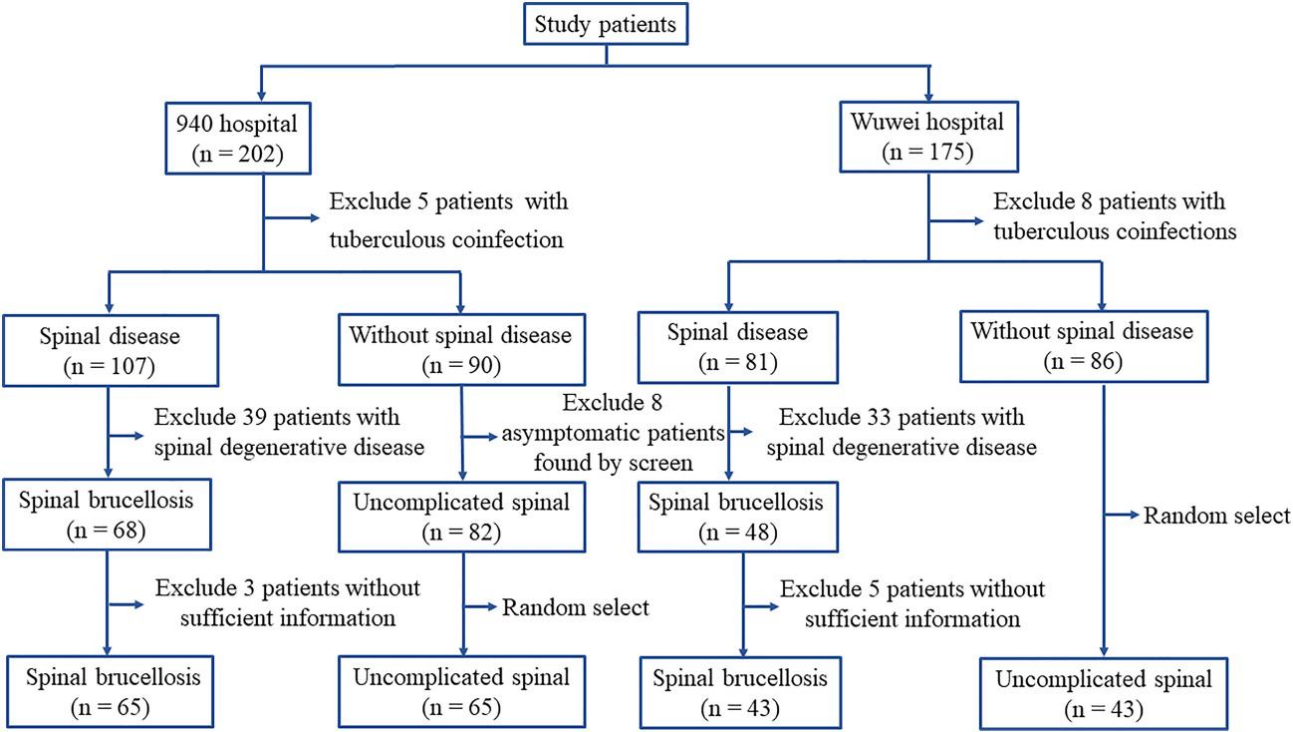
Flowchart of patient enrollment in the study.

During the study period, 377 patients, including 202 from the 940th Hospital of the Joint Logistic Support Force of PLA and 175 from Wuwei People's Hospital, received a diagnosis of brucellosis. Figure 1 illustrates the patient enrollment process. Spinal brucellosis was detected in 108 patients (28.64%).

### Epidemiological Features of Spinal Involvement in Brucellosis

**Figure 2.**
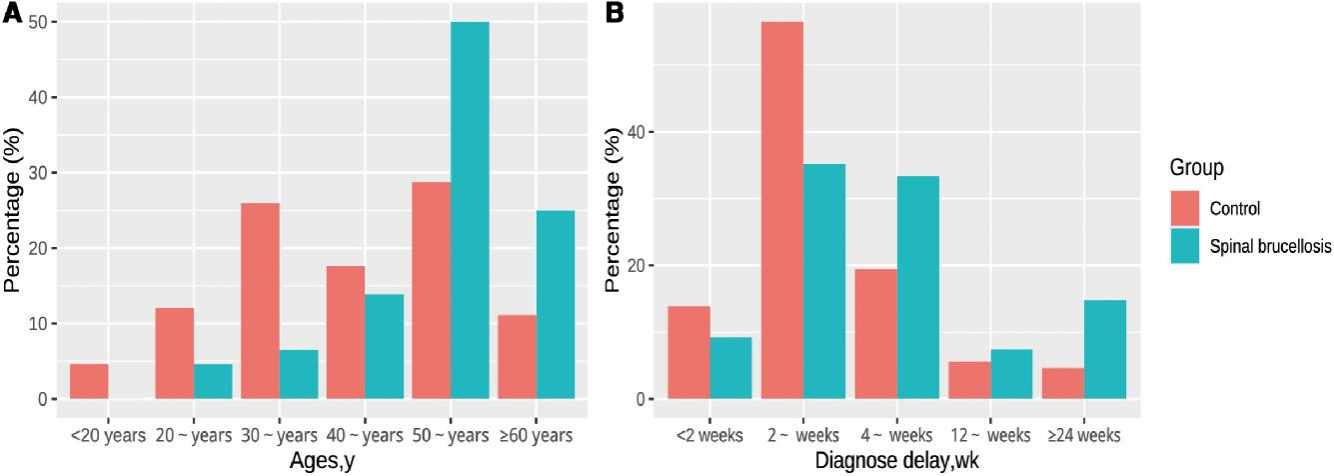
Age (*A*) and diagnostic delay (*B*) distributions in the case and control groups.

Table 1 presents the demographic characteristics of the case and control groups. The case group was significantly older (mean age [standard deviation (SD)], 53.25 [10.48] vs 43.12 [13.84] years in the control group; *P* < .001) and had a significantly longer mean diagnostic delay (11.17 [13.55] vs 6.03 [8.02] weeks, respectively; *P* = .001). Figure 2 depicts the age and diagnostic delay distributions of the case and control groups; 87.96% of the case patients were >40 years old, and 56.48% had a diagnostic delay >4 weeks. Other demographic characteristics did not differ significantly between the 2 groups.

**Table 1.** Demographic and Epidemiological Characteristics in the Case and Control Groups

Characteristic	Patients, No. (%)^a^		*P* Value
	Case Group (n = 108)	Control Group (n = 108)	
Age, mean (SD), y	53.25 (10.48)	43.12 (13.84)	<.001
Sex			
Male	82 (75.93)	87 (80.56)	.41
Female	26 (24.07)	21 (19.44)	
Ethnicity			
Han	96 (88.89)	98 (90.74)	.93
Dongxiang	5 (4.63)	4 (3.70)	
Hui	5 (4.63)	5 (4.63)	
Other	2 (1.85)	1 (0.93)	
Occupation			
Animal herder	105 (97.22)	97 (89.81)	.050
Other	3 (2.78)	11 (10.19)	
Hypertension	6 (5.56)	12 (11.11)	.14
Diabetes	6 (5.56)	12 (11.11)	.14
Coronary heart disease	2 (1.85)	1 (0.93)	>.99
Smoking	8 (7.41)	14 (12.96)	.18
Alcohol	1 (0.93)	2 (1.85)	>.99
Animal exposure			
Sheep	60 (55.56)	57 (52.78)	.68
Cattle	40 (37.04)	40 (37.04)	>.99
Family history of brucellosis infection	4 (3.70)	3 (2.78)	>.99
Clinical type			
Acute and subacute	98 (90.74)	103 (95.37)	.28
Chronic	10 (9.26)	5 (4.63)	
Time from symptom onset to diagnosis, mean (SD), wk	11.17 (13.55)	6.03 (8.02)	.001

Abbreviation: SD, standard deviation.

^a^Data represent no. (%) of participants unless otherwise specified.

### Factors Associated With Spinal Involvement

Bivariate analysis revealed that spinal involvement in brucellosis was associated with age >40 years, employment as an animal herder, and diagnostic delay >4 weeks. In the multivariate logistic regression model (Table 2), only age >40 years (OR, 5.42, [95% CI, 2.65–11.05]; *P* < .001) and diagnostic delay >4 weeks (2.94 [1.62–5.35]; *P* < .001) were found to be independently associated with spinal involvement in brucellosis.

**Table 2.** Risk Factors for Spinal Involvement in Brucellosis

Risk Factor	Patients, No. (%)		Univariate Analysis		Multivariate Analysis	
	Case Group (n = 108)	Control Group (n = 108)	OR (95% CI)	*P* Value	OR (95% CI)	*P* Value
Age						
>40 y	95 (87.96)	61(56.48)	5.63 (2.82–11.26)	<.001	5.42 (2.65–11.05)	<.001
≤40 y	13 (12.04)	47 (43.52)				
Occupation						
Animal herder	105 (97.22)	97 (89.81)	3.97 (1.07–14.65)	.050	…	…
Other	3 (2.78)	11 (10.19)				
Diagnostic delay						
>4 wk	61 (56.48)	32 (29.63)	3.08 (1.76–5.41)	<.001	2.94 (1.62–5.35	<.001
≤4 wk	47 (43.52)	76 (70.37)				

Abbreviations: CI, confidence interval; OR, odds ratio.

### Clinical Characteristics of Spinal Involvement in Brucellosis

**Figure 3.**
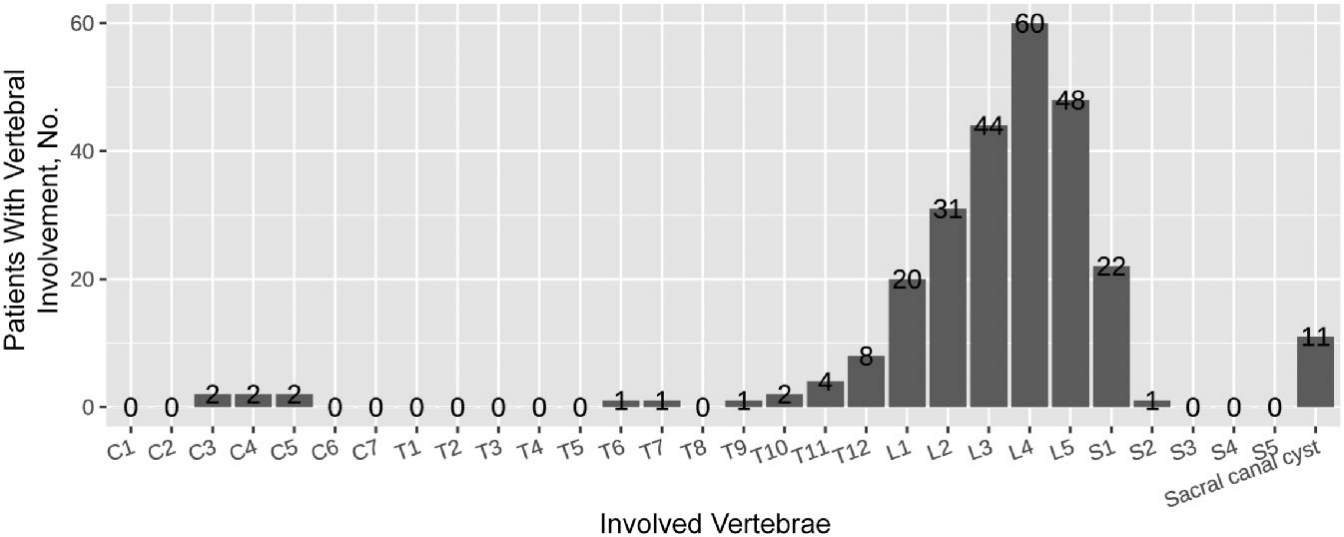
Distribution of vertebral involvement by vertebrae according to radiological imaging in spinal brucellosis.

**Figure 4.**
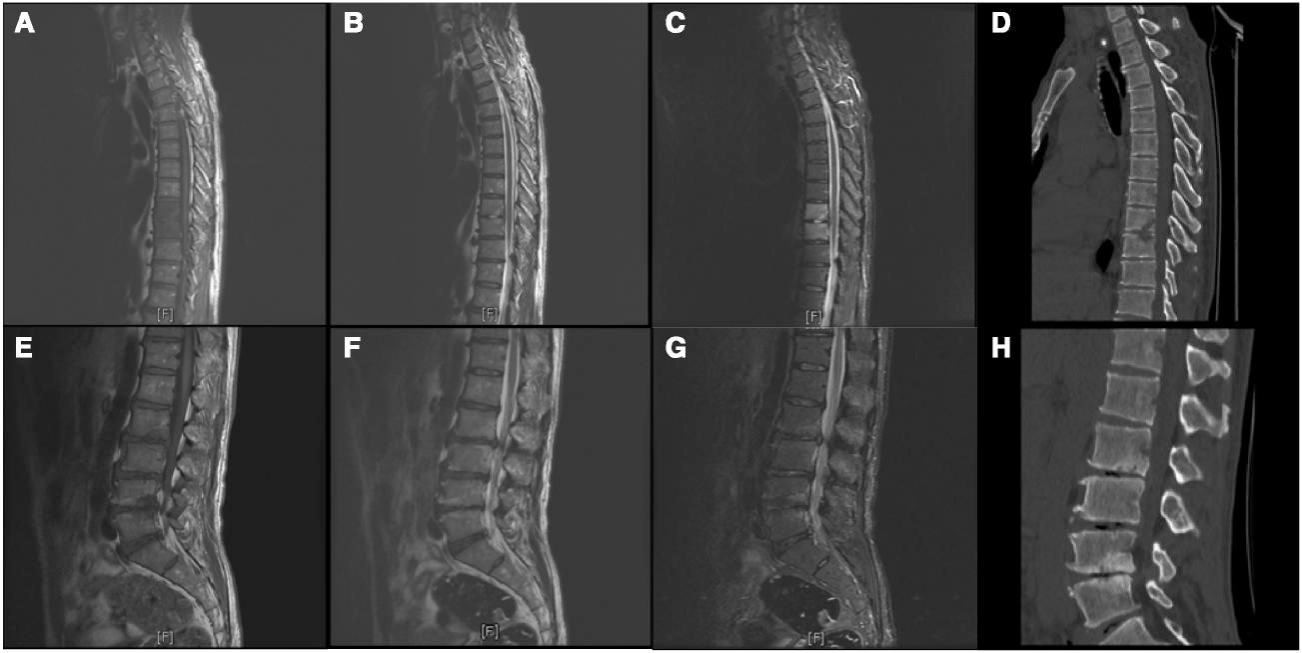
Magnetic resonance (MR) imaging and computed tomographic (CT) results on a 49-year-old man with spinal brucellosis. Sagittal T1-weighted (*A, E*) and T2-weighted (*B, F*) MR images show T9, T10, L2, L3, L4, and L5 vertebral body hypointensity and slight hyperintensity, respectively. *C, G,* T2-weighted fat-suppression images display hyperintensity in the infected vertebrae. *D, H,* CT sagittal reconstruction shows T9, T10, L2, L3, L4, and L5 vertebral body destruction and intervertebral space narrowing caused by infection.

**Figure 5.**
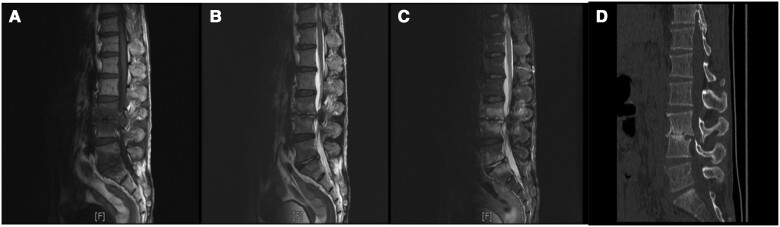
Magnetic resonance (MR) imaging and computed tomographic (CT) results in a 57-year-old man with spinal brucellosis. *A–C,* Sagittal T1-weighted (*A*) and T2-weighted (*B*) and T2-weighted fat-suppression (*C*) MR images show multiple abnormal signals in L3, L4, L5, and S1 vertebral body levels. *D,* CT sagittal reconstruction indicated L3 and L4 vertebral body destruction and intervertebral space narrowing.

Figure 3 illustrates the involvement of a single vertebral body, where the lumbar region was the most frequently involved vertebral level (203 of 249 [81.53%]), followed by the sacral (23 of 249 [9.24%]), thoracic (17 of 249 [6.83%]), and cervical (6 of 249 [2.41%]) regions. The lumbar spine was the most affected at the L3–5 level (152 of 249 [61.04%]). Spondylodiscitis complications included paravertebral abscess, psoas abscess, and sacral canal cysts or intraspinal cysts in 35 (32.41%), 13 (12.04%), and 16 (14.81%) of the patients, respectively. Figures 4 and 5 show the typical MR imaging and CT findings exhibiting spinal brucellosis. Sagittal T1- and T2-weighted images display vertebral body hypointensity and slight hyperintensity, respectively, and T2-weighted fat-suppression images show hyperintensity in the infected vertebrae.

Table 3 presents the clinical symptoms in the case and control groups. The most common complaints in the case group were back pain (n = 92 [85.19%]), fatigue (n = 64 [59.26%]), sweating (n = 62 [57.41%]), fever (n = 60 [55.56%]), and arthralgia (n = 50 [46.30%]). Back pain (92 of 108 in the case group vs 21 of 108 in the control group; *P* < .001), sweating (62 vs 46 of 108, respectively; *P* = .03), arthralgia (50 vs 67 of 108; *P* = .02), myalgia (29 of 105 vs 43 of 108; *P* = .04), cough (8 vs 18 of 108; *P* = .04), and splenomegaly (23 vs 42 of 108; *P* = .005) differed significantly between the 2 groups.

**Table 3.** Clinical Symptoms in the Case and Control Groups

Symptom	Patients, No. (%)		*P* Value
	Case Group (n = 108)	Control Group (n = 108)	
Back pain	92 (85.19)	21 (19.44)	<.001
Fatigue	64 (59.26)	70 (64.81)	.40
Sweating	62 (57.41)	46 (42.59)	.03
Fever	60 (55.56)	68 (62.96)	.27
Arthralgia	50 (46.30)	67 (62.04)	.02
Myalgia	29 (26.85)	43 (39.81)	.04
Chills	25 (23.14)	24 (22.22)	.87
Anorexia	25 (23.15)	17 (15.74)	.17
Nausea	12 (11.11)	5 (4.63)	.08
Headache	10 (9.26)	13 (12.04)	.51
Dizziness	9 (8.33)	11 (10.19)	.64
Cough	8 (7.41)	18 (16.67)	.04
Dyspnea	4 (3.70)	3 (2.78)	.70
Abdominal pain	5 (4.63)	4 (3.70)	.73
Scrotal pain	2 (1.85)	6 (5.56)	.15
Lymphadenopathy	12 (11.11)	11 (10.19)	.82
Splenomegaly	23 (21.30)	42 (38.89)	.005
Hepatomegaly	2 (1.85)	3 (2.78)	.65

Table 4 presents other complications in the case and control groups. Sixty-eight patients (62.96%) experienced other complications. The most common complications were skeletal system complications (n = 31 [28.70%]), followed by genitourinary (n = 20 [18.52%]), respiratory (n = 16 [14.81%]), gastrointestinal (n = 16 [14.81%]), and cardiovascular (n = 7 [6.48%]) complications. In the case group, renal (17 vs 5 of 108; *P* = .007) and hepatic cysts (14 vs 5 of 108; *P* = .03) were more common.

**Table 4.** Other Complications in the Case and Control Groups

Complication	Patients, No. (%)		*P* Value
	Case Group (n = 108)	Control Group (n = 108)	
Skeletal system			
Sacroiliitis	7 (6.48)	4 (3.70)	.35
Peripheral arthritis	26 (24.07)	22 (20.37)	.51
Osteomyelitis	7 (6.48)	7 (6.48)	>.99
Genitourinary system			
Orchitis	2 (1.85)	7 (6.48)	.09
Epididymitis	3 (2.78)	7 (6.48)	.20
Scrotal swelling	2 (1.85)	2 (1.85)	>.99
Renal cyst	17 (15.74)	5 (4.63)	.007
Respiratory system			
Pneumonia	9 (8.33)	10 (9.26)	.81
Pleural adhesion	12 (11.11)	9 (8.33)	.49
Pleural effusion	5 (4.63)	5 (4.63)	>.99
Cardiovascular system			
Carditis	0	1 (0.93)	>.99
Pericardial effusion	7 (6.48)	7 (6.48)	>.99
Gastrointestinal system			
Cholestasis	3 (2.78)	4 (3.70)	.70
Hepatic cyst	14 (12.96)	5 (4.63)	.03
Ascites	0	2 (1.85)	.50

Table 5 presents the laboratory features of the 2 groups. The case group had significantly higher erythrocyte sedimentation rate (mean [SD], 39.53 [29.18] vs 14.73 [18.83] mm/h in the control group; *P* < .001), platelet count (220.58 × 10^9^/L [81.90 × 10^9^/L] vs 180.92 × 10^9^/L [61.67 × 10^9^/L], respectively; *P* = .003), platelet-lymphocyte ratio (137.83 [63.05] vs 114.19 [52.99]; *P* = .048), and γ-glutamyl transferase level (57.16 [44.97] vs 37.91 [31.24] IU/L; *P* = .007) and significantly lower albumin-globulin ratio (1.17 [0.26] vs 1.30 [0.30]; *P* = .001) and serum creatinine level (57.32 [14.93] vs 65.82 [17.06] μmol/L; *P* = .004). In addition, 16 blood cultures from both groups were positive, and the isolates were identified as types of *Brucella melitensis*.

**Table 5.** Laboratory Features of the Case and Control Groups

Measurement	Laboratory Value, Mean (SD)^a^		*P* Value
	Case Group (n = 108)	Control Group (n = 108)	
Standard agglutination test, titer	1/200	1/200	.76
Erythrocyte sedimentation rate, mm/h	39.53 (29.18)	14.73 (18.83)	<.001
C-reactive protein, mg/L	26.11 (32.12)	24.85 33.94)	.85
Procalcitonin, ng/mL	0.18 (0.21)	0.36 (0.77)	.16
Interleukin 6, pg/mL	35.955 (2.26)	63.67 (209.03)	.433
Blood cell count, ×10^9^/L			
Red blood cells	4.56 (0.64)	4.63 (0.54)	.57
White blood cells	5.70 (1.65)	5.38 (2.01)	.34
Neutrophils	3.41 (1.27)	2.97 (1.67)	.11
Lymphocytes	1.75 0.62)	1.86 (0.87)	.41
Monocytes	0.45 0.18)	0.43 (0.21)	.55
Eosinophils	0.07 (0.06)	0.09 0.09)	.17
Basophils	0.02 (0.01)	0.02 (0.02)	.19
Platelets	220.58 (81.90)	180.92 (61.67)	.003
Hemoglobin, g/L	134.58 (17.51)	137.95 18.75)	.31
Neutrophil-lymphocyte ratio	2.17 (1.26)	2.11 (1.80)	.85
Platelet-lymphocyte ratio	137.83 (63.05)	114.195 (2.99)	.048
Monocyte-lymphocyte ratio	0.28 (0.14)	0.270.17)	.84
Aspartate transaminase, IU/L	25.44 (16.51)	27.10 (17.01)	.59
Alanine transaminase, IU/L	36.69 (35.81)	35.43 (32.22)	.84
Alkaline phosphatase, IU/L	100.59 (38.57)	91.94 (62.06)	.36
γ-Glutamyl transferase, IU/L	57.16 (44.97)	37.91 (31.24)	.007
Creatine kinase, umol/L	55.62 (44.64)	59.82 (37.18)	.64
Creatine kinase isoenzyme, IU/L	15.21 (22.20)	16.47 (30.19)	.81
α-Hydroxybutyric dehydrogenase, IU/L	166.48 (43.65)	198.08 (118.85)	.07
Lactate dehydrogenase, IU/L	180.375 (2.51)	216.17 (196.66)	.23
Albumin, g/L	34.08 (4.74)	35.765 (.45)	.07
Globulin, g/L	31.54 (5.17)	30.35 5 (.03)	.20
Albumin-globulin ratio	1.17 (0.26)	1.30 (0.30)	.001
Total bilirubin, μmol/L	11.68 (5.79)	13.59 (6.20)	.08
Serum creatinine, μmol/L	57.32 (14.93)	65.82 (17.06)	.004
High-density lipoprotein, mmol/L	0.76 (0.24)	0.77 (0.25)	.88
Low-density lipoprotein, mmol/L	2.24 (0.77)	2.26 (0.80)	.68
Total cholesterol, mmol/L	3.55 (1.08)	3.64 (1.07)	.72
Blood culture, no. (%)	8 (7.41)	8 (7.41)	>.99

Abbreviation: SD, standard deviation.

^a^Data represent mean (SD) values unless otherwise specified.

Moreover, 17 patients (15.74%) underwent vertebral surgery. The surgical procedure consisted of debridement and posterior pedicle screw fixation based on drug treatment. The prognosis in patients who underwent surgery did not differ significantly from that in nonsurgical cases.

## DISCUSSION

Human brucellosis is a major health concern worldwide. Spondylodiscitis is a common but serious complication of brucellosis in humans. This study describes the epidemiological and laboratory findings in patients with spinal involvement in brucellosis, as well as risk factors associated with this condition.

Research on the epidemiological characteristics of spinal brucellosis is currently limited. The present study demonstrated that patients with brucellosis and spinal involvement were significantly older than those without spinal involvement. The prognosis is likely to be unfavorable for older patients, and spondylodiscitis occurs more frequently in adults and older people than in other populations [9, 14]. In the present study, age >40 years was an independent risk factor for spinal involvement in brucellosis. Patients with brucellosis aged >40 years had a 5.42 times greater risk of spinal involvement than those aged ≤40 years.

Diagnostic delay is another reported risk factor for spinal involvement in brucellosis. In the present study, the mean diagnostic delay was significantly longer in patients with spinal involvement than in those without spinal involvement (11.17 vs 6.03 weeks, respectively). Although a previous study reported that patients with osteoarticular disease experienced a greater diagnostic delay than other patients [15], that study lacked a control group. In the present study, a diagnostic delay of >4 weeks increased the risk of developing spinal infections by 2.94 times. This risk may be explained by the higher frequency of spinal brucellosis (28.64%) in the present study compared with other studies [8–10]; moreover, the present study had a longer mean diagnostic delay than other studies in high-risk regions of China.

Owing to its nonspecific clinical signs, human brucellosis is subject to common diagnostic delays [16]. Accordingly, the diagnostic delay is multifactorial. The clinical manifestations of brucellosis are typically nonspecific and include various clinical symptoms, thereby possibly leading to misdiagnosis. A study involving 2060 cases reported that 57.62% of patients with brucellosis were misdiagnosed or suspected of having other diseases with similar clinical symptoms [17]. Delays in diagnosis also result from health service inadequacies and socioeconomic factors. The average diagnostic delay was reported to be shorter in urban areas than in villages [17]. Moreover, older age is associated with a longer diagnostic delay [16]. However, the present study determined that older age and diagnostic delay were both independent risk factors for spinal involvement in brucellosis. Therefore, the risk of spinal involvement in older adults may increase because of diagnostic delays. Patients with brucellar spondylitis appear to respond better to antibiotic therapy with optimal duration and surgical intervention when required [12]. Thus, older people should be given increased attention, and diagnostic capacity should be improved to reduce diagnostic delays and facilitate prompt and appropriate treatment.

Spondylodiscitis mostly affects the lumbosacral region [18]. In the present study, the L3–5 lumbar spine was the most commonly affected region among the enrolled patients. Localized spinal pain is the earliest sign of brucellar spondylitis, and back pain is the most common symptom in patients with spinal involvement. Furthermore, 85.9%–100% of patients with spinal involvement present with back pain [8, 19, 20]. Strengthening health education in the target population is crucial for prevention and control of brucellosis. When individuals in high-risk areas present with back pain, brucellosis should be suspected.

The present study also demonstrated that renal and hepatic cysts were more common in patients with spinal involvement, and their serum creatinine, albumin-globulin ratio, and liver transferase levels differed significantly from those of patients without spinal involvement. This finding might be linked to kidney and liver involvement in brucellosis [21, 22]. Patients with spinal involvement have higher erythrocyte sedimentation rates and platelet counts than those without spinal involvement. Elevated erythrocyte sedimentation rate and platelet count are closely associated with osteoarticular involvement [23, 24]. These 2 factors can be used to estimate the clinical course of brucellosis [25].

The present study had some limitations. For example, selection bias resulting from case and control enrollment is possible. Patient data were collected retrospectively; thus, some records of events provided by the participants might be inaccurate or incomplete. Accordingly, the bias was reduced by selecting participants from 2 hospitals, Wuwei people’s hosptial for a secondary hospital and 940th hospital for a tertiary hospital. Stratified analysis revealed that age >40 years and diagnostic delay >4 weeks were independent risk factors for spinal involvement in brucellosis among patients from both hospitals. Cohort studies are required to demonstrate these relationships as well as intervention effects and pathogenic mechanisms. The relationship between diagnostic delay and other brucellosis complications also needs further exploration.

In summary, age >40 years and diagnostic delay >4 weeks are risk factors for spinal involvement in brucellosis. The risk of spinal involvement increases as the age and diagnostic delay increase. Therefore, diagnostic delays and older age in high-risk areas should be given attention, and diagnostic delays should be shortened, using effective measures to reduce the risk of spinal involvement.

## References

[1] Lai S, Chen Q, Li Z. Human brucellosis: an ongoing global health challenge. China CDC Weekly 2021; 3:120–3. 34595017 10.46234/ccdcw2021.031 PMC8393116

[2] Qureshi KA, Parvez A, Fahmy NA, et al Brucellosis: epidemiology, pathogenesis, diagnosis and treatment-a comprehensive review. Ann Med 2023; 55:2295398. 38165919 10.1080/07853890.2023.2295398 PMC10769134

[3] Laine CG, Johnson VE, Scott HM, Arenas-Gamboa AM. Global estimate of human brucellosis incidence. Emerging Infect Dis 2023; 29:1789–97. 10.3201/eid2909.230052 PMC10461652 37610167

[4] Spernovasilis N, Karantanas A, Markaki I, et al *Brucella* spondylitis: current knowledge and recent advances. J Clin Med 2024; 13:595. 38276100 10.3390/jcm13020595 PMC10816169

[5] Esmaeilnejad-Ganji SM, Esmaeilnejad-Ganji SMR. Osteoarticular manifestations of human brucellosis: a review. World J Orthop 2019; 10:54–62. 30788222 10.5312/wjo.v10.i2.54 PMC6379739

[6] Kazak E, Akalın H, Yılmaz E, et al Brucellosis: a retrospective evaluation of 164 cases. Singapore Med J 2016; 57:624–9. 26768063 10.11622/smedj.2015163 PMC5331138

[7] Koubaa M, Maaloul I, Marrakchi C, et al Spinal brucellosis in south of Tunisia: review of 32 cases. Spine J 2014; 14:1538–44. 24331843 10.1016/j.spinee.2013.09.027

[8] Liang C, Wei W, Liang X, De E, Zheng B. Spinal brucellosis in Hulunbuir, China, 2011–2016. Infect Drug Resist 2019; 12:1565–71. 31239732 10.2147/IDR.S202440 PMC6559255

[9] Shi Y, Gao H, Pappas G, et al Clinical features of 2041 human brucellosis cases in China. PloS One 2018; 13:e0205500. 30476930 10.1371/journal.pone.0205500 PMC6258468

[10] Jiang W, Chen J, Li Q, et al Epidemiological characteristics, clinical manifestations and laboratory findings in 850 patients with brucellosis in Heilongjiang province, China. BMC Infect Dis 2019; 19:439. 31109292 10.1186/s12879-019-4081-5 PMC6528215

[11] Turgut M, Turgut AT, Koşar U. Spinal brucellosis: Turkish experience based on 452 cases published during the last century. Acta Neurochir (Wien) 2006; 148:1033–44; discussion 1044. 16944052 10.1007/s00701-006-0877-3

[12] Turgut M, Haddad FS, Oreste DD. Neurobrucellosis: clinical, diagnostic and therapeutic features. Berlin, German: Springer; 2016.

[13] Colmenero JD, Ruiz-Mesa JD, Plata A, et al Clinical findings, therapeutic approach, and outcome of brucellar vertebral osteomyelitis. Clin Infect Dis 2008; 46:426–33. 18181740 10.1086/525266

[14] Keramat F, Hashemi SH, Esna-ashari F, Kaseb K. Clinical and para-clinical features of brucellosis with and without spondylodiscitis. Avicenna J Clin Microbiol Infect 2021; 8:39–44.

[15] Zribi M, Ammari L, Masmoudi A, Tiouiri H, Fendri C. Clinical manifestations, complications and treatment of brucellosis: 45-patient study [in French]. Pathol Biol 2009; 57:349–52. 18387752 10.1016/j.patbio.2008.02.003

[16] Zhai J, Peng R, Wang Y, et al Factors associated with diagnostic delays in human brucellosis in Tongliao city, Inner Mongolia autonomous region, China. Front Public Health 2021; 9:648054. 34692615 10.3389/fpubh.2021.648054 PMC8526552

[17] Wang Y, Zhang W, Ke Y, et al Human brucellosis, a heterogeneously distributed, delayed, and misdiagnosed disease in China. Clin Infect Dis 2013; 56:750–1. 23175566 10.1093/cid/cis980

[18] Turgut M, Cullu E, Sendur OF, Güyrer G. Brucellar spine infection—four case reports. Neurol Med Chir (Tokyo) 2004; 44:562–7. 15633472 10.2176/nmc.44.562

[19] Ulu-Kilic A, Karakas A, Erdem H, et al Update on treatment options for spinal brucellosis. Clin Microbiol Infect 2014; 20:O75–82. 24118178 10.1111/1469-0691.12351

[20] Solera J, Lozano E, Martínez-Alfaro E, Espinosa A, Castillejos ML, Abad L. Brucellar spondylitis: review of 35 cases and literature survey. Clin Infect Dis 1999; 29:1440–9. 10585793 10.1086/313524

[21] Akritidis N, Tzivras M, Delladetsima I, Stefanaki S, Moutsopoulos HM, Pappas G. The liver in brucellosis. Clin Gastroenterol Hepatol 2007; 5:1109–12. 17482524 10.1016/j.cgh.2006.08.010

[22] Jin M, Fan Z, Gao R, Li X, Gao Z, Wang Z. Research progress on complications of brucellosis. Front Cell Infect Microbiol 2023; 13:1136674. 37065189 10.3389/fcimb.2023.1136674 PMC10102637

[23] Aktug-Demir N, Kolgelier S, Ozcimen S, Sumer S, Demir LS, Inkaya AC. Diagnostic clues for spondylitis in acute brucellosis. Saudi Med J 2014; 35:816–20. 25129179

[24] Sen P, Demirdal T, Nemli SA. Predictive value of inflammation markers in brucellosis. Arch Iran Med 2019; 22:640–5. 31823629

[25] Okan DH, Gökmen Z, Seyit B, Yuksel K, Cevdet Z, Deniz A. Mean platelet volume in brucellosis: correlation between *Brucella* standard serum agglutination test results, platelet count, and C-reactive protein. Afr Health Sci 2014; 14:797–801. 25834485 10.4314/ahs.v14i4.4 PMC4370056

